# 1.1-µm Band Extended Wide-Bandwidth Wavelength-Swept Laser Based on Polygonal Scanning Wavelength Filter

**DOI:** 10.3390/s21093053

**Published:** 2021-04-27

**Authors:** Gi Hyen Lee, Soyeon Ahn, Jinhwa Gene, Min Yong Jeon

**Affiliations:** 1 Department of Physics, College of Natural Sciences, Chungnam National University, 99 Daehak-ro Yuseong-gu, Daejeon 34134, Korea; gtit5de@naver.com (G.H.L.); ahnsoyen5@naver.com (S.A.); 2 Institute of Quantum Systems (IQS), Chungnam National University, 99 Daehak-ro Yuseong-gu, Daejeon 34134, Korea; genejh@gmail.com

**Keywords:** wavelength-swept laser, fiber lasers, semiconductor optical amplifier, dynamic measurement, dynamic optical fiber sensors

## Abstract

We demonstrated a 1.1-µm band extended wideband wavelength-swept laser (WSL) that combined two semiconductor optical amplifiers (SOAs) based on a polygonal scanning wavelength filter. The center wavelengths of the two SOAs were 1020 nm and 1140 nm, respectively. Two SOAs were connected in parallel in the form of a Mach-Zehnder interferometer. At a scanning speed of 1.8 kHz, the 10-dB bandwidth of the spectral output and the average power were approximately 228 nm and 16.88 mW, respectively. Owing to the nonlinear effect of the SOA, a decrease was observed in the bandwidth according to the scanning speed. Moreover, the intensity of the WSL decreased because the oscillation time was smaller than the buildup time. In addition, a cholesteric liquid crystal (CLC) cell was fabricated as an application of WSL, and the dynamic change of the first-order reflection of the CLC cell in the 1-µm band was observed using the WSL. The pitch jumps of the reflection band occurred according to the electric field applied to the CLC cell, and instantaneous changes were observed.

## 1. Introduction

A wavelength-swept laser (WSL) is a light source that can continuously change its narrow linewidth wavelength over a wide wavelength range at high speed [1,2,3,4,5,6,7,8,9,10,11,12,13,14,15,16,17,18,19]. Owing to its wide wavelength band and fast wavelength scanning speed, it is primarily used as a light source for optical coherence tomography (OCT) systems in biophotonics [2,3,4,5,6,7,8,9,10,11,12]. In addition, the output of the WSL has a one-to-one correspondence in the spectral and temporal domains; thus, it has been widely applied as a light source for dynamic fiber optic sensors that measure dynamic changes in wavelength [13,14,15,16,17,18]. WSLs can be implemented using a variety of methods. Among them, the polygonal scanning wavelength filter-based WSL [2,3,4,17,18,19,20] and the Fabry-Perot tunable filter (FFP-TF)-based WSL [5,6,7,8,9,10,11,12,13,21,22,23] have been studied most actively. In addition, an electro-optical tunable filter (EOTF) [24] and acousto-optic tunable filter (AOTF) [25,26] have been researched. FFP-TF has the advantage of optical alignment, which can be easily implemented because all elements comprise a pigtailed optical fiber; however, the center wavelength of the filter is unstable, owing to thermal instability. Because a sinusoidal function is applied to the filter, the signal is nonlinear and requires complex signal processing [10,11]. An EOTF is not limited by a mechanical drive and fast reaction speed, and it operates linearly [24]. Therefore, the scanning bandwidth is small and the linewidth is relatively wider than that of other methods. An AOTF also has the advantage of no mechanical drive; however, the bandwidth of the maximum gain is not sufficiently wide and the scanning speed is slow [25,26]. Although the polygonal scanning wavelength filter is relatively bulky and is limited by mechanical driving, it is possible to easily change the scanning speed and scanning wavelength range by adjusting the rotation speed, diffraction grating angle, and the magnification of the telescope [2,3,4,17,18,19,20].

Most WSLs have been implemented for biophotonics imaging applications in the 1300-nm band [2,3,4,5,8,9,10,11,12]. In addition, many studies have utilized various wavelength bands, such as 850- [24,27,28,29], 1000- [30,31,32], 1500- [6,13,17,21,22,25,33,34,35], and 1700–2000-nm [36,37,38] bands, as light sources for OCT imaging, including optical fiber sensors. Research on WSLs typically focuses on obtaining a fast scanning speed [15,39,40,41,42,43,44] and scanning in a wide wavelength band [8,9,12,37] to improve imaging quality or the sensing dynamic range. MEMS-based WSL [45,46], dispersion tuned WSL [15,47,48,49], very short cavity WSL using FFP-TF [9], and Fourier domain mode locked WSL [5,6,11,12,13,16,22] were implemented to achieve a fast scanning speed. Methods to implement a wide scanning wavelength band include connecting two gain media in parallel and using a semiconductor optical amplifier (SOA) with a wide gain area [4,8,12]. If a wide scanning wavelength band in a WSL is implemented in the 1-µm band, high resolution can be realized as an OCT light source or the dynamic measurement range can be increased in the optical fiber sensor system.

In this study, we successfully demonstrated, for the first time to our knowledge, a >228-nm wideband WSL around 1.1-µm band based on a polygonal scanning wavelength filter using two SOAs. Two SOAs were combined in parallel as a Mach-Zehnder interferometer in the laser cavity. This enabled a wider wavelength scanning band by combining the adjacent wavelength bands of the two SOAs. In addition, the characteristics of the scanning bandwidth and average power were investigated with respect to the scanning speed of the WSL. As an application of the dynamic measurement of WSL, the phenomenon of the pitch jump was observed according to the intensity of the electric field applied to a cholesteric liquid crystal (CLC) cell and the observation of the instantaneous movement of the first-order reflection band on the oscilloscope was reported.

## 2. Experiments

The WSL is a wavelength-tunable laser that continuously and rapidly varies with time in a wide wavelength bandwidth. In the wavelength-tunable filter inserted into the laser cavity, only the maximum gain corresponding to the filter condition is fed back to the resonator when an amplified spontaneous emission (ASE) beam with a wide bandwidth is incident on the filter. By continuously changing these conditions, the WSL continuously oscillates over a wide bandwidth.

**Figure 1:**
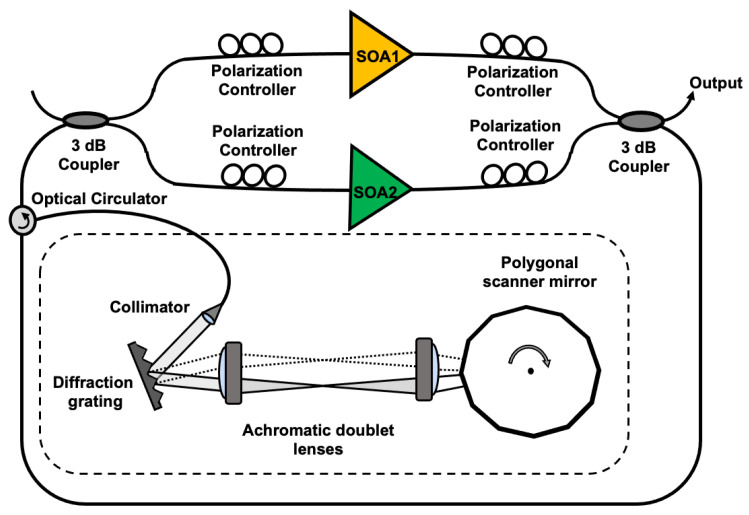
Schematic diagram of an experimental setup, in which two SOAs were connected in parallel in the form of a Mach-Zehnder interferometer.

Figure 1 shows a schematic diagram of the experimental setup, in which two SOAs were connected in parallel in the form of a Mach-Zehnder interferometer to construct a single polygonal scanning wavelength filter-based WSL. This obtained a wider wavelength scanning band by combining the adjacent wavelength bands of the two SOAs [4,8,12]. When two SOAs are connected in series, a wide scanning band cannot be obtained because the gain of one SOA is absorbed by the other [4]. The broadband WSL consisted of two SOAs, two polarization controllers in front and behind each SOA, two 50:50 fiber couplers, an optical circulator, and a polygonal scanning wavelength filter; the last is indicated by the dotted box in Figure 1. The polygonal scanning wavelength filter contained a brazed diffraction grating, a telescope with two lenses, and a 36-facet polygonal scanning mirror. The telescope was comprised of two achromatic doublet lenses with a grating at the front focal plane of the first lens and a polygonal scanning axis on the back focal plane of the second lens. The parallel beam from the collimator was incident on a brazing diffraction grating and underwent diffraction of the first order, which was incident on the telescope and aligned along the optical axis. The diffracted wavelength components had different angles of convergence on the polygonal scanning mirror facet. Therefore, the polygonal scanning mirror only reflected the spectral components within a narrow resolution band that were vertically incident. The reflected wavelength component was fed back into the laser cavity. Because the polygonal scanning mirror rotated at a high speed, the lasing wavelength continuously varied within the gain band. If the polygonal scanning mirror was rotated in the direction of increasing wavelength, the energy was transmitted in a long wavelength, owing to the nonlinear effect of the SOA. Therefore, a higher output and narrower line width was obtained, compared with the opposite case. A 600-lines/mm diffraction grating was used, the angle of incidence was 47°, the angle of reflection was 3.5°, and the focal lengths of the two lenses were each 5 cm. The output from the WSL was monitored on an oscilloscope using a photodetector and an optical spectrum analyzer (OSA).

**Figure 2:**
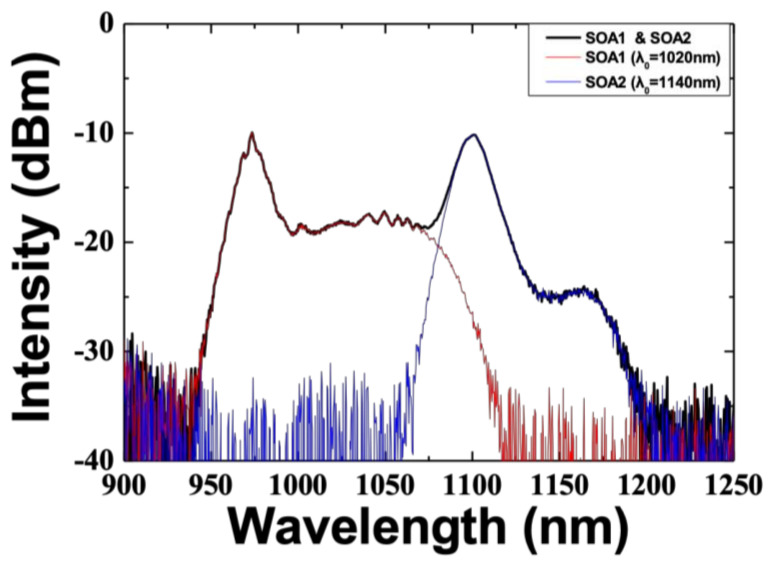
ASE spectra of two SOAs.

In the experiment, a 1064 ± 100-nm broadband optical fiber coupler and a broadband circulator operating at 950–1100 nm were used to minimize the optical loss across the wide wavelength band. The center wavelengths of the ASE of SOA 1 and SOA 2 were 1020 nm and 1140 nm, respectively, and the 10-dB bandwidths were 114 nm and 57 nm, respectively, as shown in Figure 2.

**Figure 3:**
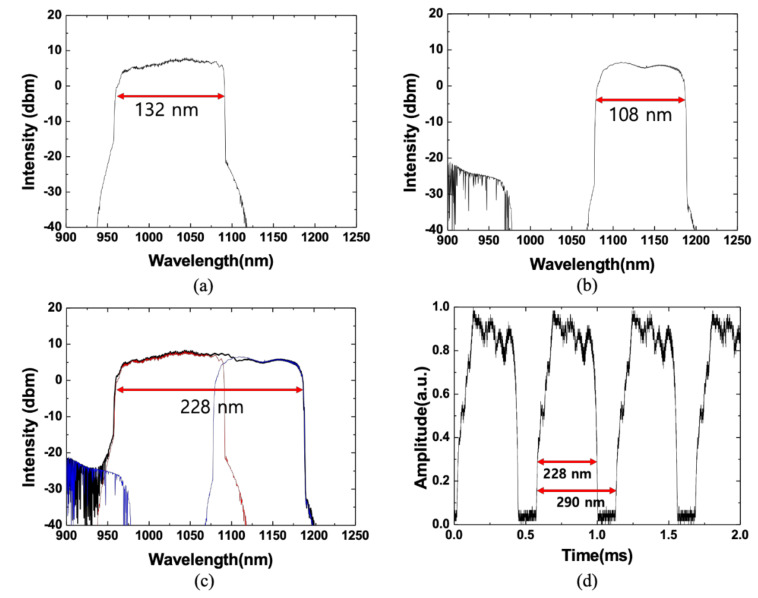
(**a**) Optical spectrum of the WSL when only SOA 1 was connected in the Mach-Zehnder interferometer; (**b**) optical spectrum of the WSL when only SOA 2 was connected in the Mach-Zehnder interferometer; (**c**) optical spectra output and (**d**) corresponding temporal output from the WSL by combining two SOAs in parallel in the Mach-Zehnder interferometer.

Figure 3a shows the optical spectrum of the WSL when only SOA 1 was connected in the Mach-Zehnder interferometer. The 10-dB bandwidth and average output power were 132 nm (from 959 nm to 1091 nm) and 11.46 mW, respectively. Figure 3b shows the optical spectrum of the WSL when only SOA 2 was connected in the Mach-Zehnder interferometer. The 10-dB bandwidth and average output power were 108 nm (from 1079 nm to 1187 nm) and 4.73 mW, respectively. Figure 3c shows the optical spectra output from the fabricated WSL by combining the two SOAs in parallel at a scanning speed of 1.8 kHz. The red and blue lines denote the optical spectrum when only SOA 1 or SOA 2 were connected to the Mach-Zehnder interferometer of the laser cavity, respectively. The optical spectrum of the WSL, obtained by connecting the two SOAs together, is represented as a black line. The 10-dB bandwidth and the average output power of the WSL with two SOAs were ~228 nm (from 959 nm to 1187 nm) and ~16.88 mW, respectively. The wavelength scanning resolution measured by OSA was 2 nm, but the same scanning bandwidth of 228 nm or more was obtained with 0.2-nm resolution. This is a significantly wider scanning bandwidth compared with that of the ASE with two SOAs, and the output spectrum of the WSL achieved a relatively uniform amplitude. This can be achieved by controlling the pump current of each SOA and controlling the polarization appropriately using the polarization controllers in the laser cavity. The instantaneous linewidth was determined to be 0.11 nm in this laser cavity. Figure 3d shows the output of the WSL measured using the oscilloscope. This corresponds to the optical spectrum of the WSL, shown in Figure 3c. In the oscilloscope, the time interval for a scanning bandwidth pulse was measured by 440 µs, and a period was measured as 560 µs. The period corresponds to free spectral range (FSR) of the wavelength filter. By inversely converting the wavelength scanning range of 228 nm corresponding to the time interval of 440 µs of the scanning bandwidth pulse, the wavelength range corresponding to 560 µs was inversely estimated. The obtained FSR was approximately 290 nm. The measurement error was ~2.5% due to the resolution of the oscilloscope. The FSR of the wavelength filter can be calculated using the following equation [2,50]:

(1) $${\left(\Delta \lambda \right)}_{\mathrm{FSR}\;}=p\;\left(\cos\;\beta_{0}\right)\frac{F_{2}}{\bar{F_{1}}}\theta_{0\;},$$

where *p* is the grating pitch, *θ*_0_ = 2π/36 is the facet-to-facet polar angle of the polygonal scanner mirror, *β*_0_ is the angle between the optical axis of the telescope and the grating normal, and *F*_1_ and *F*_2_ are the focal lengths of the two lenses in the telescope. Using Equation (1), the calculated FSR of the wavelength filter is 290 nm. The FSR measured on the oscilloscope was approximately 290 nm, which is similar to the theoretical value. The measured 10-dB wavelength scanning range of the WSL was ~228 nm; therefore, the duty cycle was approximately 78.6%. In order to obtain the desired FSR and to operate normally, it is necessary to appropriately adjust various variables shown in Equation (1). However, in order to obtain sufficient wavelength scanning, the FSR must be greater than the wavelength scanning band of the light source.

**Figure 4:**
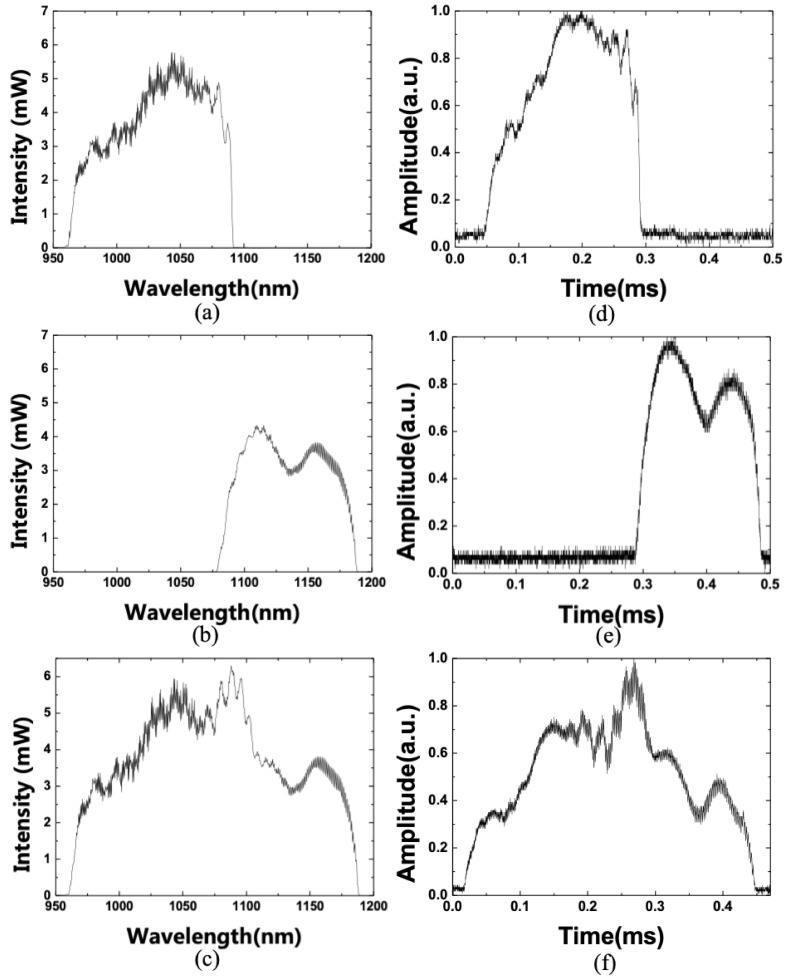
Optical spectrum of WSL with a linear scale (**a**) when only SOA 1 was connected, (**b**) when only SOA 2 was connected, and (**c**) when SOA1 and SOA2 were combined in a Mach-Zehnder interferometer configuration. (**d**–**f**) Output pulses in the temporal domain corresponding to (**a**–**c**).

Figure 4a,b shows the optical spectra of WSL with a linear scale when only SOA 1 or SOA 2 were independently connected, and Figure 4c shows the optical spectrum of the WSL with a linear scale when SOA1 and SOA2 were combined in a Mach-Zehnder interferometer configuration. If the output spectra of the two SOAs overlapped, the interference between the two laser outputs can cause intensity noise. However, in the case of a parallel configuration, beating noise may occur if the resonator lengths have exactly matched each other, but it can be eliminated by introducing an offset of the length between the two arms within the Mach-Zehnder interferometer [4,12]. Additionally, Figure 4d–f shows the output pulses in the temporal domain corresponding to Figure 4a–c. They show a one-to-one correspondence between the shape of the pulse signal in the temporal domain and the wavelength band in the spectral domain. Therefore, the dynamic optical properties of a material can be inferred from a wavelength signal by measuring the output pulse in the temporal domain using the WSL.

**Figure 5:**
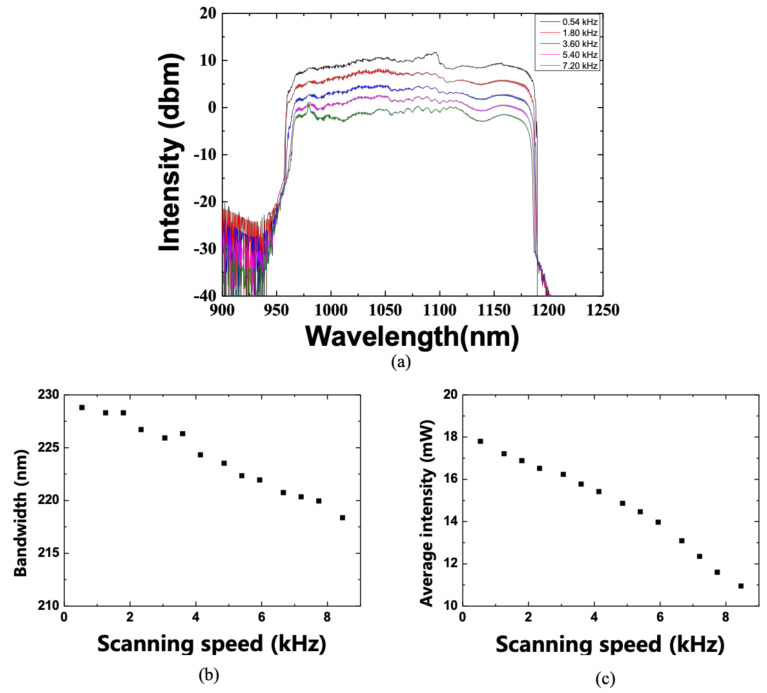
(**a**) Optical spectra, (**b**) 10-dB bandwidth, and (**c**) average optical power with respect to the WSL scanning speed.

To investigate the characteristics of the WSL, the change in the scanning bandwidth was measured while increasing the scanning speed. Figure 5a shows the variation in the spectra according to the scanning speed of the WSL. Up to 2 kHz, a 10-dB scanning bandwidth of the WSL achieved over ~228 nm. As the scanning speed increased, the scanning bandwidth gradually decreased. At approximately 8 kHz, the scanning bandwidth was reduced to ~220 nm, as shown in Figure 5b. Similarly, as shown in Figure 5c, as the scanning speed increased, the average optical output power gradually decreased. This occurred because the gain was not sufficiently obtained at an oscillation time smaller than the buildup time of the WSL.

**Figure 6:**
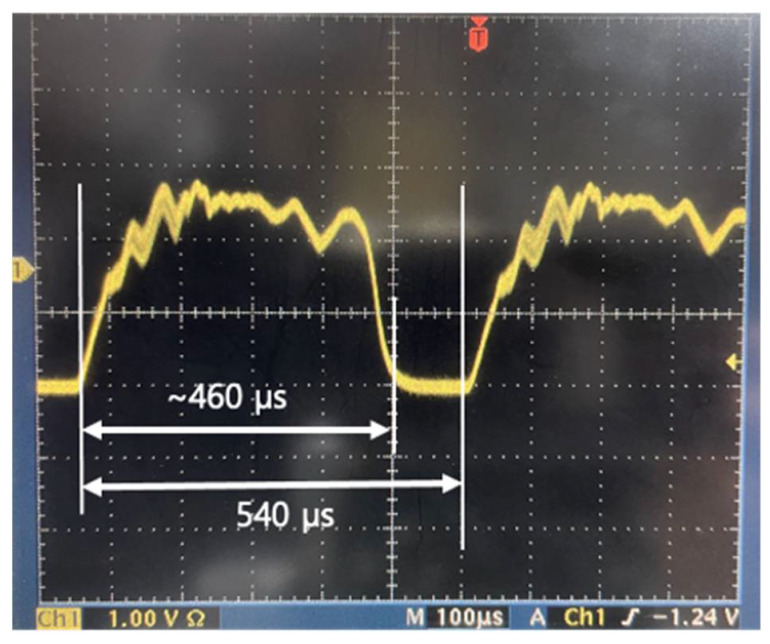
Photograph of the output signal of the WSL on an oscilloscope.

As mentioned above, the output of WSL has a one-to-one correspondence in the spectral and temporal domain; therefore, it has been widely applied as a light source to measure dynamic changes in wavelength [13,14,15,16,17,18]. As a simple application, the dynamic variation of the first-order reflection spectrum from a CLC cell is measured by applying an electric field to the CLC cell in the 1-µm band. In the experiments, a nematic liquid crystal E7 and chiral dopant (R811) were mixed to produce a right-handed CLC. The chiral dopant concentration of the CLC cell was 13.92 wt%, and the calculated pitch was 678 nm. Figure 6 shows a photograph of the output signal of the WSL on an oscilloscope. The scanning speed was 1.8 kHz and the duty cycle was ~78.6%. The time intervals of 440 µs and 560 µs on the oscilloscope correspond to a 228-nm scanning bandwidth and a 290-nm FSR in the spectral domain, respectively.

**Figure 7:**
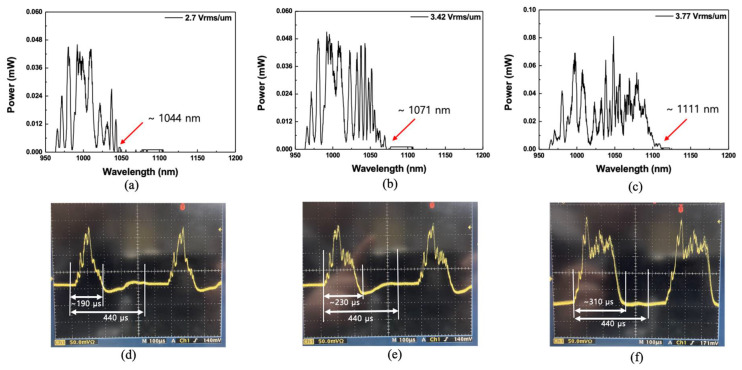
Optical spectra of the first-order reflection band from the CLC cell when the electric field was (**a**) 2.70, (**b**) 3.42, and (**c**) 3.77 V_rms_/µm, and (**d**–**f**) the oscilloscope displays of the first-order reflection band corresponding to the applied electric fields in (**a**–**c**), respectively [Supplementary Videos S1 and S2].

The beam from the WSL was incident on the CLC cell, and the transmission spectra for the first-order reflection with respect to the intensity of the electric field applied to the CLC cell is observed. Figure 7a–c shows the optical spectra of the reflection band when the applied electric field is 2.70, 3.42, and 3.77 V_rms_/µm, respectively. The wavelength of the arrow indicated in the figures is the wavelength of the short-band edge of the reflection band. When an electric field of 2.70 V_rms_/µm or more was applied to the CLC cell, the short edge of the reflection band shifted to 1044 nm from 1022 nm. When the electric field applied to the CLC cell increased, the reflection band moved to longer wavelength discontinuously, owing to the pitch jump, which occurred instantaneously [19]. When an electric field of 3.42 V_rms_/µm or more was applied, the short edge shifted to 1071 nm, as shown in Figure 7b. Because the OSA’s response to the wavelength change was slow, it was not easy to observe the abrupt change in the reflection band spectrum, owing to the instantaneous pitch jump. However, when the dynamic variations of the WSL were observed in the temporal domain, using an oscilloscope and high-speed photodetector, the process of changing the wavelength of the reflection band owing to the instantaneous pitch jump could be observed. Figure 7d–f shows the oscilloscope displays of the first-order reflection band when the applied electric field was 2.70, 3.42, and 3.77 V_rms_/µm, respectively. These correspond to Figure 7a–c, respectively. On the oscilloscope, if the electric field applied to the CLC cell was continuously increased, it could be observed in real time that the short-band edge of the reflection band was instantaneously moved by the pitch jump at any moment. As an example, when the electric field increased from 3.42 to 3.77 V_rms_/µm, the pitch jump occurred in the CLC cell. Supplementary Material Videos S1 and S2 show videos of the in situ variation of the first-order reflection band spectrum, owing to the dynamic pitch jump of the CLC cell, on the oscilloscope. Supplementary Material Video S1 shows a video in which the reflection band changes when the electric field applied to the CLC cell increases from 3.42 to 3.77 V_rms_/µm and Supplementary Material Video S2 shows the electric field applied to the CLC cell is increased from 3.77 to 4.13 V_rms_/µm.

**Figure 8:**
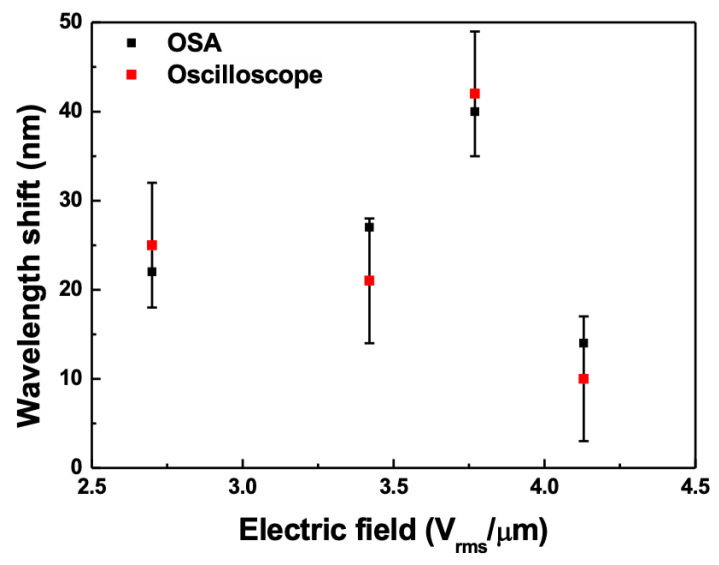
Wavelength shift of the short-band edge on the OSA and oscilloscope when a pitch jump occurs according to the electric field applied to the CLC cell.

Figure 8 shows the wavelength shift of the short-band edge on the OSA and oscilloscope when a pitch jump occurred with respect to the electric field applied to the CLC cell. In the figure, the error bar represents the error owing to the thickness of the line when measured using the oscilloscope. It can be observed that the wavelength shifts obtained by converting the time interval measured on the oscilloscope into a wavelength are notably consistent within the measurement error compared with that measured by the OSA. Therefore, if a WSL is used to measure the dynamic wavelength change, it can also easily determine the wavelength change in the OSA by measuring the waveform change using the oscilloscope and converting it into a wavelength.

## 3. Conclusions

We successfully demonstrated a wide-bandwidth WSL based on a polygonal scanning wavelength filter using two SOAs. By combining two SOAs in parallel in the form of a Mach-Zehnder interferometer, we achieved a 10-dB bandwidth of ~228 nm (from 959 to 1187 nm). We also investigated the changes in the scanning bandwidth and average output power with respect to the scanning speed of the WSL. The bandwidth and average power of the WSL decreased because the oscillation time was smaller than the buildup time of the SOA. A CLC cell was fabricated to investigate the first-order reflection band, owing to the dynamic pitch jump, with respect to the electric field applied to the CLC cell in the 1100-nm band region. The instantaneous change in the reflection band of the CLC cell was due to the instantaneous pitch jump of the CLC. Moreover, the dynamic change in the reflection band of the CLC cell was confirmed by converting the instantaneous change of the waveform on the oscilloscope into the corresponding wavelength change. The wide scanning wavelength band in the 1.1-µm band of WSL is expected to be used as a high-resolution OCT light source or to increase the dynamic measurement range in a fiber optic sensor system.

## Data Availability

Data available on request from the authors.

## Supplementary Materials

The following are available online at https://www.mdpi.com/1424-8220/21/9/3053/s1. Video S1: A video in which the reflection band changes when the electric field applied to the CLC cell increased from 3.42 V_rms_/µm to 3.77 V_rms_/µm. Video S2: A video in which the reflection band changes when the electric field applied to the CLC cell increased from 3.77 V_rms_/µm to 4.13 V_rms_/µm.
